# Challenges in modeling complexity of neglected tropical diseases: a review of dynamics of visceral leishmaniasis in resource limited settings

**DOI:** 10.1186/s12982-017-0065-3

**Published:** 2017-09-18

**Authors:** Swati DebRoy, Olivia Prosper, Austin Mishoe, Anuj Mubayi

**Affiliations:** 1 Department of Mathematics and Computational Science, University of South Carolina, Beaufort, SC USA; 2 Department of Mathematics, University of Kentucky, Lexington, KY USA; 3 Simon A. Levin-Mathematical Computational and Modeling Science Center, School of Human Evolution and Social Change, Arizona State University, Tempe, AZ USA

**Keywords:** Mathematical model, Dynamical modeling, Kala-azar, Leishmaniasis, Risk-factors

## Abstract

**Objectives:**

Neglected tropical diseases (NTD), account for a large proportion of the global disease burden, and their control faces several challenges including diminishing human and financial resources for those distressed from such diseases. Visceral leishmaniasis (VL), the second-largest parasitic killer (after malaria) and an NTD affects poor populations and causes considerable cost to the affected individuals. Mathematical models can serve as a critical and cost-effective tool for understanding VL dynamics, however, complex array of socio-economic factors affecting its dynamics need to be identified and appropriately incorporated within a dynamical modeling framework. This study reviews literature on vector-borne diseases and collects challenges and successes related to the modeling of transmission dynamics of VL. Possible ways of creating a comprehensive mathematical model is also discussed.

**Methods:**

Published literature in three categories are reviewed: (i) identifying non-traditional but critical mechanisms for VL transmission in resource limited regions, (ii) mathematical models used for dynamics of Leishmaniasis and other related vector borne infectious diseases and (iii) examples of modeling that have the potential to capture identified mechanisms of VL to study its dynamics.

**Results:**

This review suggests that VL elimination have not been achieved yet because existing transmission dynamics models for VL fails to capture relevant local socio-economic risk factors. This study identifies critical risk factors of VL and distribute them in six categories (atmosphere, access, availability, awareness, adherence, and accedence). The study also suggests novel quantitative models, parts of it are borrowed from other non-neglected diseases, for incorporating these factors and using them to understand VL dynamics and evaluating control programs for achieving VL elimination in a resource-limited environment.

**Conclusions:**

Controlling VL is expensive for local communities in endemic countries where individuals remain in the vicious cycle of disease and poverty. Smarter public investment in control programs would not only decrease the VL disease burden but will also help to alleviate poverty. However, dynamical models are necessary to evaluate intervention strategies to formulate a cost-effective optimal policy for eradication of VL.

**Electronic supplementary material:**

The online version of this article (doi:10.1186/s12982-017-0065-3) contains supplementary material, which is available to authorized users.

## Introduction

Visceral leishmaniasis (VL) is a vector-borne infectious disease that is transmitted to humans by infected sandflies and is the second-largest parasitic killer in the world after malaria [1, 2]. If left untreated, most cases result in death within 2–3 years of clinical manifestation [3, 4]. In 2015, more than 90% of new cases of VL reported to the World Health Organization (WHO) occurred in Brazil, Ethiopia, India, Kenya, Somalia, South Sudan, and Sudan [2]. VL is identified as a Neglected Tropical Disease (NTD) by the WHO because it is endemic in several poverty stricken regions of the world [5], although preventive measures and successful treatment is common in most developed countries [6]. Many people living in these impoverished regions are daily-wage workers, for whom infection with a disease like VL restricts the bread-winners’ ability to provide livelihood for their families. Moreover, the cost of treatment and duration of stunted income pushes them into a vicious cycle of further hardship and irrecoverable financial deprivation. Although local government authorities and the WHO have devised several control programs (such as integrated vector management program and active surveillance) to lower the burden of VL in these regions [7], the VL endemicity always creeps back after a brief period of relief. This ineffectiveness has been attributed to several factors, including severe under-reporting of cases and death due to VL, lack of clarity in the etiology of the disease (example, contribution asymptomatic infections have on transmission is not known [8]), and limited estimation of reservoirs of the infection (example, distribution of post-kala-azar dermal leishmaniasis is unknown [9]). Thus, the intensity and extent of the control programs have been in conflict with the magnitude of the true VL burden. In such resource-limited regions mathematical models can help shed light on respective contribution of several of these challenges to the VL endemicity (including identifying cost-effective driving mechanisms), as it has done for other infectious diseases like malaria. Hence, immediate attention from the modeling community is in dire need.

In the past, the WHO has set several elimination target dates for VL for years 2010 and 2015, which could not be achieved in the Indian subcontinent [10]. The primary reason for this shortcoming may be the ineffective implementation of policies in the under-developed regions affected by VL. Mathematical modeling approaches in conjunction with model guided additional field research in India could be a turning point for achieving optimal program implementation and may help to (1) quantify the “true” burden of VL in Bihar where it has proven to be particularly difficult to eliminate, (2) investigate the potential mechanisms for the spread of the Leishmania parasite, and (3) suggest optimal vector control programs that may help in achieving the WHO goal of elimination of VL by the year 2017 [11]. The target of the VL elimination program is to reduce the annual incidence to less than 1 per 10,000 at the district or sub district level in South Asia by 2017 [12–14]. However, the incidence in some parts may be around 20 cases per 10,000 [12, 14, 15].

Since Sir Ronald Ross’ first paper using a mathematical model to study the transmission dynamics of malaria in 1906, there have been many modeling studies focusing primarily on vector-borne disease [16]. Existing studies of VL and other NTDs have been either limited in their understanding of the transmission dynamics of the disease or fail to capture critical local socio-economic factors. Hence, more studies are needed on neglected tropical diseases such as VL, clarifying unknown aspects of etiology and modeling based long term evaluation of dynamical interventions. Our main objective in this study is to suggest mathematical modeling approaches for capturing identified regional issues that may be critical in better evaluating control programs; thereby, developing optimal cost-effective intervention strategy and timely achieving elimination goals. We discuss specific features of VL (including treatment availability, living conditions, effect of social status, and implementation cost of control programs) that should be incorporated into quantitative methods. We assessed the strengths and limitations of analytical models in addressing the risk factors and VBD transmission. Finally, we provide recommendations for future research on modeling VL dynamics and impact of risk factors on VL transmission.

## Methods

### Search strategy

The following protocol was established for this review. We searched Google Scholar, PubMed and Web of Knowledge to find articles focusing on “mathematical modeling of vector-borne diseases” (VBD), “Visceral Leishmaniasis”, “Kala-azar”, “risk factors of Leishmaniasis”, and “dynamics of Leishmaniasis”. The goal was to identify factors that have been modeled and studied for understanding VL dynamics and inform modeling strategies that needs to be undertaken in order to systematically address challenges for controlling (eventually eliminating) VL from resource limited regions. For the theoretical literature, we included epidemiological and public health studies that addressed important technicalities of modeling the dynamics of vector borne infectious diseases, including, economic models, uncertainty and sensitivity analysis, and optimization models. We also considered studies that evaluate impact of socio-economic conditions on patterns of tropical diseases.

### Selection criteria

Our search criterion was: articles that contain visceral leishmaniasis (VL) AND VBD model AND risk factors of VL. Our eligibility criteria were articles that: (i) were published in peer reviewed journals and focused on countries affected with neglected tropical diseases; (ii) aimed to model risk factors for similar (to VL) vector-borne diseases; (iii) employed mechanistic models to understand VL dynamics; and (iv) establish critical risk factors from cross-sectional and longitudinal empirical VL (in South Asia) studies. A scheme of the selection in outlined in a flowchart in the Additional file 1: Figure S4. To identify data-supported risk factors, we reviewed the literature based on searches using the term visceral leishmaniasis with the subheading risk factors, and the geographic terms India or Bangladesh or Nepal. Articles published from 1985 through September, 2016 in English were included. We included all studies that explicitly addressed factors associated with risk of VL in South Asia, the objectives, design, outcome measures and analyses were clearly described, and judged to be adequate based on the methods and data presented. A formal comparison for studies on risk factors was impossible due to scarce data from different time points, and non-comparable study designs and implications. We excluded studies that did not incorporate disease transmission dynamics such as studies with cost-effectiveness, and decision tree type models. We also excluded opinion papers, review articles, qualitative VL reports and modeling studies comparing different regions. More details on how many articles were retrieved by the search and how many were excluded on each topic were given in first paragraph of Results section.

## Results

We collected 107 articles on topics mentioned in the Search Strategy section (Additional file 1: Figure S4). Among these 6 articles (out of 11 resulted from key-word search)were on general epidemiological description of family of leishmaniasis other than visceral leishmaniasis and 3 articles (out of 7) were on other vector borne diseases not similar to VL. Hence, these articles were not incorporated in the review. A total of 65 articles were studied on risk factors of visceral leishmaniasis, however, 43 articles were eventually chosen for review based on the consistent findings of risk factors within them and our selection criteria. The risk factors in these 43 articles were grouped under six categories: Atmosphere (20/31 articles were selected), Access (6/9), Availability (4/7), Awareness (4/5), Adherence (7/10) and Accedence (2/3). The 24 articles found in the databases are on the dynamics of related (to Leishmaniasis) vector borne diseases while 12 articles present interesting results on transmission dynamics of Leishmaniasis. Three additional articles on dynamics of Leishmaniasis were not incorporated in the review as their primary goal was mathematical analysis rather than implications for the Leishmaniasis.

### Identified VL risk factors: challenges for leishmaniasis transmission dynamics in resource-limited regions

**Fig. 1:**
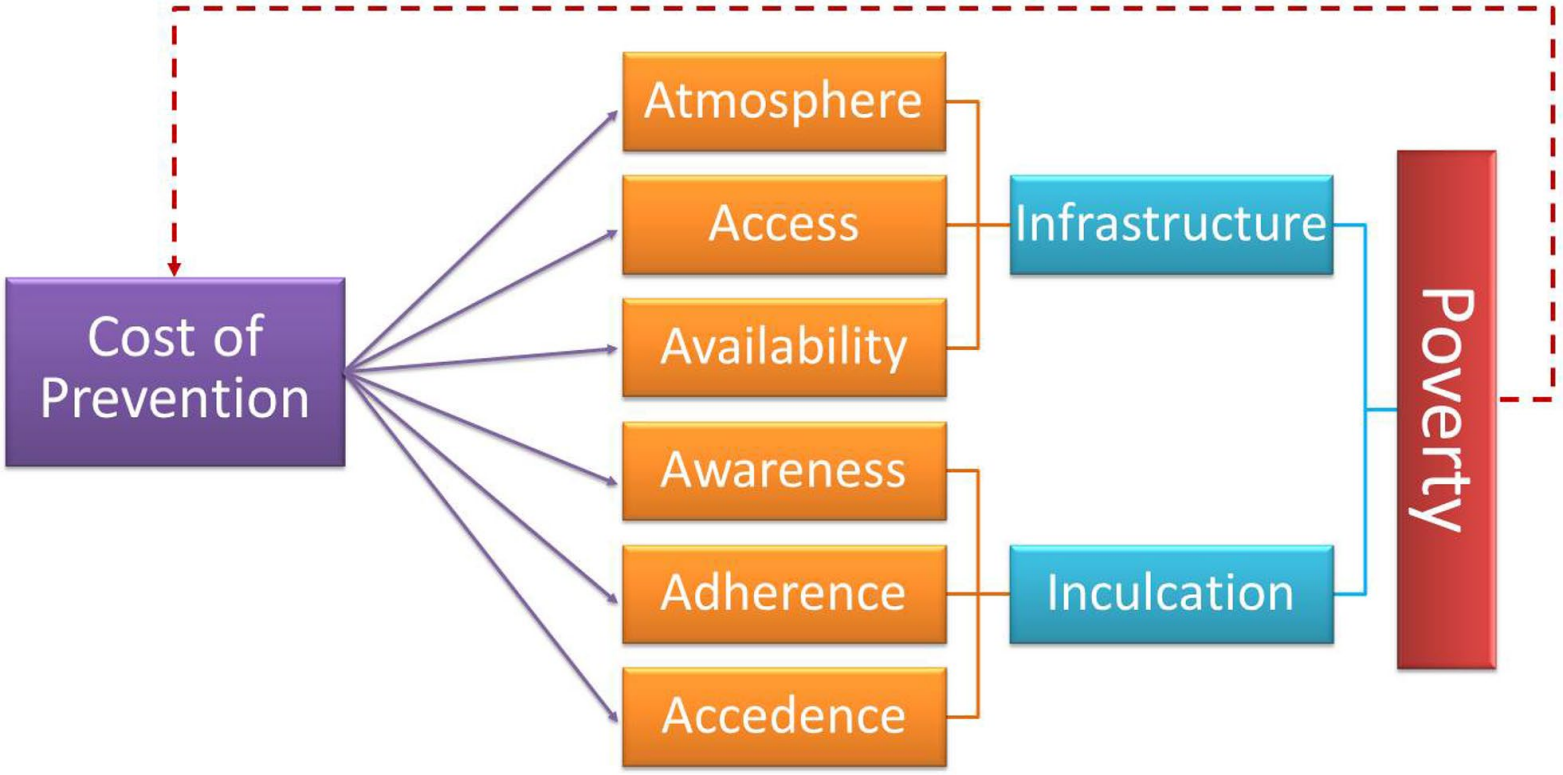
A vicious cycle of socio-economic challenges and difficulty to access disease interventions

The depth and complexity of the socio-economic challenges of a neglected tropical disease dynamics at the grassroots level may seem disparate when considering them individually. Thus, to better comprehend the nature of obstacles in the transmission process, we classified some of the key issues into the six categories viz., atmosphere, access, availability, awareness, adherence and accedence (6 A-s). We note that these 6 As in turn can be grouped into either inculcation or infrastructure (2 I-s) (Fig. 1). Endemicity of VL in the region is fueled by the deficiency in the 2 I-s, which in turn stems from the sheer poverty in the affected area. Ultimately, it is the poverty which cannot bear the cost of prevention, and leads to a vicious cycle of VL dynamics there. In this review we focus on the state of Bihar, which holds around 80% of India’s VL cases [17], and its neighboring countries of Nepal and Bangladesh where the disease dynamics are similar. We proceed to describe the relevance of this 6 A-s. Some key features defining the 6 A-s are represented in Fig. 1.

#### Atmosphere

In the state of Bihar, the worst affected areas are in remote agricultural villages. Studies have revealed several living conditions positively correlated with higher prevalence of leishmaniasis. In two independent studies, factors like mud plastered houses, vegetation and bamboo near the house, and granary inside the house were found to significantly contribute to leishmaniasis [18, 19]. Other systematic studies in the subcontinent with similar geographical settings (Uttar Pradesh, India; West Bengal, India; Terai, Nepal; Mymensingh, Bangladesh) have found that living conditions with cracked mud walls, damp floors, and close proximity to a water body are risk factors for leishmaniasis [20]. Also, a high density of occupants in a household with more than three people per room were found to increase transmission [19, 21–28].

It is well known that sand-fly bites thrive during the warmer months (March–June, October in India), and late in the evening [29]. The role of climatic factors on transmission dynamics of vector-borne diseases has been thoroughly studied in the literature [30, 31]. The hot Indian summer in combination with lack of electricity often lead people to sleep outdoors, which increases the number of sand-fly bites and hence the risk of contracting leishmaniasis [23, 25]. Understandably, proper use of bed-nets have been found to have a protective effect on people across several studies, and sleeping on a cot (versus on the floor) also demonstrated a protective effect as well [21, 23, 24, 26–28]. Proximity to domesticated animals is found to play a complex role in containing, spreading and serving as a possible reservoir of the parasite [32]. For example, some studies found that proximity to livestock provided a protective effect against leishmaniasis as livestock get bitten more instead of humans [24, 25], whereas in Uttar Pradesh, India, the risk of leishmaniasis was found to increase with increased numbers of cattle in the vicinity of a household because of large number of sandflies were attracted to cattle sites than to other livestock sites [23].

#### Access

Currently, therapeutic interventions for Kala-azar (Indian VL) are significantly subsidized by the Ministry of Health in India (National Vector Bourne Disease Control Program’s Kala-azar Elimination Initiative under the Govt. of India). In spite of the subsidize, estimates suggest that 80% of the outpatients and 57% of the inpatients are handled in the private sector (The World Bank report, 2001 [33]). Non-Government Organizations (NGOs) in Bihar usually provide awareness and education programs, carryout research, and provide access to regular health services. Ninety percent of Bihar’s population lives in rural areas where less than 1% of health services are provided by not-for-profit NGOs (The World Bank, 2001 [33]). Patients in rural areas travel on average much further for treatment than patients in urban areas. Thus, access to healthcare can be tricky at present and efforts need to be made to encourage the set-up of temporary mobile clinics in harder to reach areas and to encourage people to seek out certified treatment [3]. Bihar is the poorest state in India, where the “caste” (proxy for social standing) of a person is born into affects almost every aspect of the social conduct he/she receives their entire life. [34] Martinez et al. found that the people of lower caste are consistently being seen by a doctor at a more advanced stage of VL than those of a higher caste [35]. In fact, according to the last article, most VL patients in the disadvantaged caste see a doctor more than eight weeks post symptom onset, which includes a larger spleen and lower hemoglobin level than normal. Thus, efforts need to focus more on the people of lower caste to diminish the disparity in healthcare; only then can planned control measures effectively reduce the overall burden of VL.

#### Availability

The WHO recommends the use of a single dose of Amphotericin B as the first line of treatment in the Indian sub-continent [36]. However, daily injections of Pentavalent Antimonials (SSG) for 20–30 days and 15 injections of Amphotericin B every other day are still more widely used in India (NVBDCP). The availability of drugs in a timely manner is dependent on several factors including the affordability of a drug by the government, reasonable forecasting of the quantity of drug required (to avoid shortage as well as waste), proper storage and distribution of the drugs throughout the lengthy route from the manufacturer to the affected people, avoiding cheaper counterfeit drugs and also drug legislation [37].

#### Awareness

**Fig. 2:**
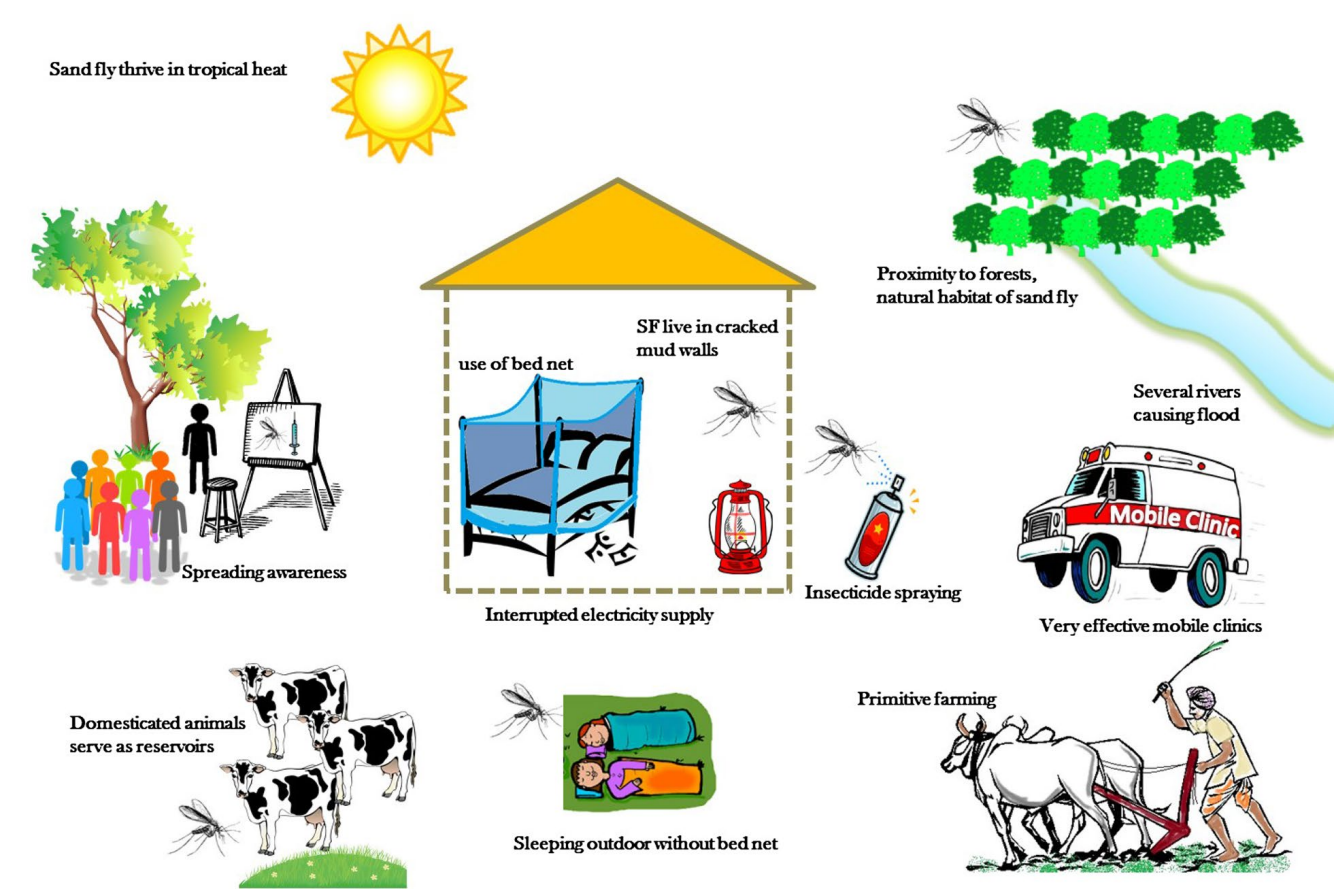
Cartoon reflecting social aspects on which awareness programs to control spread of VL can be designed to reduce disease burden

‘Awareness’ can be defined as knowledge regarding the etiology of the disease which would help local individuals to prevent infection and to look out for VL symptoms and seek medical attention sooner rather than later. Figure 2 shows some social aspects for which awareness programs may be needed as a prevention for VL. Lack of awareness causes a disease which is curable upon treatment, to end up causing death. Even symptomatic VL-infected people mix in the population freely, thus considerably increasing the chances of transmission. In a study on Nepal (which shares a border with Bihar) by Rijal et al. [38] it was found that affected people from the poorest strata of the community preferred to visit a private doctor or local faith healer over public health clinics, leading to lower short term cost but higher long term costs for these individuals because of inconsistent diagnosis and under par treatment. Moreover, debts acquired during this period, in addition to lack of income (the earning adult being ill), creates a major financial abyss which is almost impossible to recover from. Thus, it is not sufficient that the government provides free treatment to the people, it is also necessary to disseminate that information in an effective manner to every strata of people in the affected region. In a study in Brazil, awareness was spread in communities through educating school children, who in turn were assigned to discuss interventions mentioned in student’s homework assignments with their family members [39]. This intervention improved awareness significantly.

#### Adherence

Non-adherence to treatment is a major factor contributing to the high development of resistance to pentavalent antimonials in the population exposed to VL in the Indian sub-continent [37]. This resulted in discontinuing it as a first line of treatment for VL. There are several major factors which contribute to non-adherence in the region including being male, older than 35 years of age, lack of knowledge about the consequences of incomplete treatment leading to patients stopping treatment once the symptoms are relieved, and the financial loss due to reduced days of productivity while on therapy [40, 41]. It has been well documented in a 2000–2010 cohort study in Nepal that another disease, Post Kala-azar Dermal Leishmaniasis (PKDL), a sequel and reservoir for VL, was more common in patients who were inadequately treated during VL versus the ones who adhered to the full course of treatment. A 29.4% PKDL prevalence was observed in patients who were inadequately treated for VL with sodium stibogluconate (SSG) versus 2.0% PKDL prevalence in VL patients who completed the full SSG course [42]. As observed by Rijal et al. [38] loss of productivity implies no income at all for the poor families. Farmers are unable to attend to their fields, possibly during very important farming phases which results in lowered income for a considerable period of time. A lot of people in the poorest section of society in Bihar are also daily-wage earners and each missed day of work might present dire consequences for the entire family. To survive this period, the family takes out loans from private lenders at high interest, ultimately leading them to further poverty.

#### Accedence

‘Accedence’ is defined as the acceptance to undergo testing and treatment for PKDL, which can occur in patients who have recovered from VL post-treatment caused by the same pathogen. As reported by Desjeux et al. in 2013, patients with PKDL serve as a reservoir of VL and it is unlikely that VL can be eradicated without addressing the issue of diagnosing and treating PKDL [43]. In fact, aggressive measures are required to encourage people to consult a doctor in case of any persistent skin lesions, and once diagnosed these should be treated with Amphotericin B, which has been found effective in the Indian sub-continent. As reported by Thakur et al., attempts were made to fast-track diagnosis and treatment of PKDL in several badly affected districts of Bihar and yielded a very positive outcome [44]. However, these efforts need the support of the local governmental authorities to continue and succeed where it is most needed. It is evident that aggressive control measures are necessary to address every issue and to ultimately alleviate the burden of VL from Bihar. However, several of the issues arise from limited resources in the affected region and thus the control strategies should be carefully weighed by importance and cost-effectiveness [45]. Moreover, it is not only important to take drastic measures using the one-time funds generated for this purpose, but it is essential that powerful and sustainable changes in the system are established through easy and systematic ways of approaching the difficulties, which can only be attained by identifying critical mechanisms of the system. Mathematical models are one of the best methods to cheaply and systematically identify driving factors.

### Review of leishmaniasis dynamical modeling studies

Despite the incalculable harm and countless challenges leishmaniasis inflicts on populations around the globe, only a handful of publications address the problems from a mathematical modeling perspective. In fact, a recent review by Rock et al., which tabulates all mathematical models of VL, found only twenty-four articles using mathematical models for VL, several of which used the same base model structure [46]. Of these twenty-four articles, only seven addressed VL in the Indian subcontinent. Arguably, one of the greatest modeling challenges is the limited understanding of the leishmania pathogen, the sandfly vector, and how disease manifests in humans. Dye et al. [47] spear-headed the application of mathematical models to leishmania dynamics. The authors developed a simple discrete-time model with *Susceptible*, *Infected*, and *Resistant* humans to study the mechanism behind inter-epidemic periods observed in VL cases between 1875 and 1950 in Assam, India. Counter to the existing theory of the time, the model demonstrated that the observed inter-epidemic patterns could be explained by intrinsic factors in leishmania transmission. This modeling effort also stressed the significance of PKDL and sub-clinical infections in determining whether a region will have endemic or epidemic leishmaniasis. A few years later, Hasibeder et al. [48] published a compartmental delay-differential equation model for canine leishmaniasis. This model accounts for two types of dogs: those that will develop symptoms, and those that will remain asymptomatic, following infection by a sandfly. The model also explicitly describes the infection dynamics of the sandfly vector and considers a fixed delay representing the extrinsic incubation period. The authors take a heuristic approach to derive a formula for the basic reproduction number $R_0$, the number of secondary sandfly infections resulting from a single infected sandfly, in an otherwise fully susceptible population. Although this model addresses two important aspects of the natural history of the disease that may be extended to human VL, namely the asymptomatic human and infected vector populations, the model does not consider the asymptomatic population to be an infectious reservoir, assumes constant human and vector population sizes, and omits the effects of seasonality. The model does, however, introduce heterogeneous biting, determined by a dog’s “occupation”. The mathematics developed in [48] was applied to age-structured serological data for the dog population in Gozo, Malta in [49], and provided estimates for $R_0$. This modeling work was extended in [50] to include zoonotic transmission, that is, humans, dogs, and sandflies, were explicitly modeled. Dye conducted a sensitivity analysis to determine which of three control measures would be most effective in decreasing disease prevalence. Their results suggest vector control and vaccination of the human or dog population would be more effective than treating or killing infected dogs. However, in their model, they assumed that any treatment of dogs results in full recovery and the efficacy of vaccination is extremely high. These assumptions may not be completely realistic.

More recently, Stauch et al. developed a more comprehensive model of VL for the Indian subcontinent [51], and later extended it to include drug-resistant and drug sensitive *L. donovani* parasites, with a focus on Bihar, India [52]. The model proposed by Stauch et al. [51] extended the basic susceptible-infectious-recovered (SIR) model structure for the human population by segmenting the infected stage into five distinct stages according to an individual’s infection status determined by the results of three diagnostic markers. These diagnostic markers were (1) PCR, the earliest infection marker, (2) DAT, which measures antibody response, and (3) LST, which can detect cellular immunity. The model also includes treatment of symptomatic VL cases, treatment failure, relapse characterized as PKDL, PKDL treatment, and HIV-co-infection (described in their Additional file 1: Figure S4). The sandfly population is modeled using an SEI (*Susceptible-Latent-Infectious*) model. Treatment of VL is divided into first and second-line treatment, and treatment-induced mortality caused by drug-toxicity is considered. The model was fit to data from the KalaNet trial using Maximum Likelihood. The authors explored several intervention strategies, including treatment, active case detection, and vector control. Although the authors warn that their model assumes homogeneous transmission, ignoring possible clustering of cases within affected households, their modeling approach and parameter estimation argues that the large asymptomatic reservoir precludes the ability for a treatment-only control program to attain the desired target of less than 1 case per 10,000 individuals annually. Vector-based control is much more promising, but the authors estimate it can only reasonably reduce VL incidence to 18.8 cases per 10,000. Consequently, the authors emphasize the need for active case detection, effective treatment of PKDL, and vector control to achieve VL elimination.

Based on the model in Stauch et al. [51], and following up on their finding that treatment of VL does little to reduce transmission, Stauch et al. investigated the uncertainty in their parameter estimates and explored the efficacy of different vector-based control measures [53]. They estimated that $R_0$ for VL is approximately 4.71 in India and Nepal, and that reducing the sandfly population by 79% via reduction of breeding sites, or reducing the sandfly population by 67% through increasing sandfly mortality, are both sufficient to eliminate the *L. donovani* parasite in the human population. The authors argue that recent evaluations of IRS (indoor residual spraying) efficacy suggest that elimination should be possible, with the caveat that the situation may change if insecticide resistance emerges. However, vector management using LLIN’s (long-lasting insecticide-treated nets) or EVM (environmental management) would not be sufficient to achieve elimination. The authors emphasize the need to study infection rates, the parasite dynamics in both the human and vector population, animals serving as alternate hosts or potentially infectious reservoirs, and the contribution of the asymptomatic population. Furthermore, Stauch et al. suggest extensions of the deterministic modeling framework to include heterogeneity in population and seasonality.

Stauch et al. [52] extended the model from their previous study [51] to include both drug-resistant and drug-sensitive parasites. The authors considered five mechanisms by which the fitness of the resistant strain may differ from the sensitive strain: (1) increase probability of symptoms, (2) increase parasite load, (3) increase infectivity of asymptomatic humans, (4) increase transmission from symptomatic and asymptomatic host to vector, (5) increase transmission from vector to host. Simulations of this extended model indicate that a treatment failure rate over 60% is required to explain observations in Bihar. Furthermore, observations in Bihar cannot be explained without assuming an increase in fitness in resistant parasites. The authors explain that it is more likely that the necessary additional fitness is transmission-related rather than disease-related. Unfortunately, their results also suggest that once a more fit resistant parasite has been introduced, that parasite will eventually exclude the sensitive parasite, even in the absence of treatment.

The work of Mubayi et al. is the first to use a rigorous, and dynamic model to estimate underreporting of VL cases at the district level in Bihar, India [3]. The authors designed a staged-progression model, composed of a system of nonlinear, coupled, ordinary differential equations. In a typical SIR type epidemic model, the inbuilt assumption is that an individual stay in each infection category for an exponentially distributed waiting time. The stage-progression model considers a series of same infection category (for examples, $I_1, I_2,\ldots ,I_n$ for infectious category *I*), each with same average waiting rate. It exploits the fact that the sum of *n* independent exponential distributions with rate parameter $\lambda$ is a gamma distribution with shape parameter *n* and scale parameter $1/\lambda$ ($\Gamma (n,1/\lambda )$), which helps in capturing the observed variability in waiting time in a epidemiological category such as incubation period, infectious period, and treatment duration. Furthermore, the authors address the differences between public and private health care facilities in their treatment and reporting practices by assuming a fraction of infected individuals *p* seek treatment in public health care facilities, and the remaining proportion seek treatment in private clinics. Building an empirical distribution for this parameter *p* and deriving a relationship between model parameters and monthly reported incidence data allowed the authors to estimate the degree of underreporting for each district for the years 2003 and 2005. This model analysis informed by incidence data revealed that districts previously designated as low-risk areas for VL are actually likely to be high-risk: the true burden masked by underreporting.

ELmojtaba et al. presented a more classical approach to modeling VL, with a focus on Sudan, in [54–56]. Because leishmaniasis in the Sudan is zoonotic, the authors included the dynamics of an animal (rodents and dogs) reservoir in [54]. This baseline model is extended in [55] to address parasite diversity, and in [56] to explore the potential impact of mass vaccination in the presence of immigration.

More recently, Sevá et al. [57] developed a mathematical model for human and canine zoonotic VL in Brazil. The focus of this study was to test the efficacy of existing canine-based VL prevention and control methods: insecticide-impregnated dog collars, culling, and vaccination. By optimizing each of these control strategies in an ordinary differential equation model, while accounting for their respective costs, the authors were able to recommend a 90% coverage of the dog population with insecticide-impregnated collars as a control strategy that is easy to adopt and could, over time, eliminate VL in the region. All of these modeling efforts (summarized in Table 1) have either contributed to our understanding of VL or highlighted the need for better data to construct and validate future models of VL. However, there are currently no models, to the best of our knowledge, that attempt to link socio-economic factors, like the 6 A’s discussed in “Identified VL risk factors: challenges for leishmaniasis transmission dynamics in resource-limited regions” section, to VL disease transmission.

**Table 1:** Modeling studies, included in the review, that considers local risk factors and the transmission dynamics of diseases

Reference	Study area (period)	Disease	Data	Model type	6 A’s Addressed
Dye [47]	Assam Province, India (1875–1950)	Anthroponotic VL (*L. donovani*)	Epidemic data from Rogers (1908) [69], McCombie Young (1924) [70], and Sen Gupta (1951) [71]	Discrete-time compartmental model	$\times$
Hasibeder [48]	$\times$	Canine leishmaniasis, *L. infantum*	$\times$	ODEs	$\times$
Dye [49]	Gozo island in Malta (June–July 1989)	Canine leishmaniasis, *L. infantum*	cross-sectional survey including age-structured serological data	Used results from ODEs in [48]	$\times$
Dye [50]	Tropical America, Mediterranean, and China	Canine and human zoonotic VL, *L. infantum*	Cohort study of dogs (Unpublished data, Quinell RJ, Courtenay O, and Dye C) and estimates from [49]	ODEs	$\times$
Stauch [51]	India, Nepal, Bangladesh (2006–2008)	Anthroponotic VL - *L. donovani*	KalaNet project (ClinicalTrials,gov NCT00318721)	ODEs	$\times$
Stauch [52]	Bihar, India (1980–1997)	anthroponotic VL	Treatment failure rate of antimonial treatment obtained from review of clinical trials [72]	ODEs	$\times$
Stauch [53]	India, Nepal, Bangladesh (2006–2008)	Anthroponotic VL	KalaNet project	ODEs	$\times$
Mubayi [3]	Bihar, India (2003–2005)	Anthroponotic VL	Monthly incidence from 21 districts	Staged-progression model	$\times$
ELmojtaba [54–56]	Sudan	Zoonotic VL	Parameter estimates from literature	ODE	$\times$
Sevá [57]	Brazil (approx. 1990s and 2000s)	Canine and human zoonotic VL, *L. infantum*	Parameters taken from published studies, oral communication, or assumed	ODEs	$\times$
Aparicio [58]	United States	TB	U.S. and Massachusetts Census data and Parameter estimates from literature	ODEs and an age-structured PDE model	Atmosphere
Lipsitch [59]		TB		Stochastic-deterministic hybrid model	Adherence
Mason [61]	United States (approx. 1995–2004)	Type II diabetes	Electronic Medical Records, Administrative medical and pharmacy claims data, and Healthcare Effectiveness Data	Discounted Markov Decision Process	Adherence
Hallet [62]	Zimbabwe (1980s–2000s)	HIV	HIV prevalence and sexual behaviour surveillance data	ODEs and a Bayesian Melding framework	Awareness
Mushayabasa [63]	$\times$	Hepatitis C	Epidemiological data from literature	ODEs	Awareness
Fenichel [64]				Economic behavioral model/SIR	Awareness

x in the Table indicate absence of the information related to column heading

### Models of infectious diseases incorporating socio-economic risk factors

In this section, we provide some examples of published mathematical modeling studies (summarized in Table 1) where researchers have attempted to incorporate some of the factors mentioned above and studied their role in the transmission dynamics of infectious and physiological diseases. Existing models of visceral leishmaniasis, though limited in number as compared to diseases like malaria and dengue, have incorporated some of the biological complexity, contributing to a more developed understanding of the disease. However, to formulate applicable control measure recommendations with cost-estimation, models which can simultaneously incorporate the discussed risk-factors explicitly would be necessary. Many of the techniques to incorporate these factors individually can be drawn from the literature regarding heavily studied diseases like HIV, malaria, and tuberculosis [58–60].

#### Modeling atmosphere

In a simplistic mathematical model we can incorporate several risk factors associated with ambience implicitly through the interpretation and calculation of the model parameters. For example, the transmission parameter can be considered as a product of the predominant type of housing, density of vegetation around houses, number of domesticated animals, and number of inhabitants in a house [58].

#### Modeling adherence

Lipsitch et al. addressed adherence to treatment and its role in promoting drug resistance in a mathematical model for tuberculosis (TB) in the presence of bacterial heterogeneity [59]. To model non-compliance to drug therapy, the authors assumed that non-compliant patients adhere to the treatment regimen when bacterial loads are above a certain threshold, and will halt treatment when bacterial loads fall below this threshold, that is,

$$\begin{aligned} Adherence\_level(t) = \left\{ \begin{array}{ll} \frac{\alpha B(t)}{K+B(t)} &{}\quad if \; B(t)\ge N_{min} \\ 0 &{}\quad if \; B(t)< N_{min} \end{array}\right. \end{aligned}$$

where *B*(*t*) is the bacterial load at time *t* and $N_{min}$ is the threshold minimum bacterial load under which drug treatment is discontinued. The parameter *K* is critical bacterial load where adherence will become one-half and $\alpha$ is the rate at which the individual adhere to treatment per unit of bacterial load. Simulation and analysis of their stochastic-deterministic hybrid model illustrated that non-compliance is one mechanism that can give rise to bacteria resistant to one or more drugs in a multi-drug therapy. Furthermore, the authors noted that the pattern of resistance driven by non-compliance more closely resembled observations of patients on multi-drug therapy, compared with the pattern of resistance promoted by bacterial heterogeneity. Consequently, the model suggests that non-compliance plays a larger role than heterogeneity of the bacteria population in promoting resistance during multi-drug therapy. The authors noted that an exception to this pattern occurred in HIV-positive TB patients. The modeling assumption for non-compliance in this TB model addresses one of the ‘adherence’ concerns discussed in “Identified VL risk factors: challenges for leishmaniasis transmission dynamics in resource-limited regions” section, namely that patients often stop treatment once symptoms are relieved, suggesting a possible framework in which to study adherence to treatment, treatment failure, and if tied to a population-level model, the spread of drug resistant parasites in VL-endemic regions.

Adherence to treatment is also a concern in diabetes patients, despite the fact that non-adherence increases the likelihood for stroke and other potentially fatal complications. Mason et al. [61] developed a Markov Decision process (MDP) model to study the timing of treatment initiation and drug-adherence in diabetes patients and the role these two factors play in determining a patients’ quality-adjusted life years (QALYs). Consistent with observations of adherence behavior in diabetes patients, the model assumed that a patient’s health status does not influence future adherence. This assumption may be relevant for some VL-endemic regions where non-adherence is a consequence of insufficient inculcation of the risks associated with improper treatment, or a result of the cost of treatment. The model also assumed, consistent with clinical practice, that if a patient or the patient’s physician had not already decided to begin treatment, treatment would be immediately initiated following a non-fatal complication. The reward function, dependent on adherence, included several important factors, including QALY, the cost of treatment and hospitalization, and disutility resulting from treatment side effects. The authors also developed a cost function, with the goal of optimizing treatment initiation, in the presence of different degrees of non-adherence, and compared the optimal timing for ‘uncertain adherence’ and ‘predictable adherers’. The authors quantified the benefit of treatment relative to the cost through a reward function $r_t(l,m)$, where *l* denoted the patient’s health status, and *m* equaled zero or one, depending on whether the patient was on treatment or not.

$$\begin{aligned} r_{t}(l,m)&= R(l,m)-C_{t}^{0} - (CF^{S}(l)+CF^{CHD}(l)) \\& \quad - mC^{ST}(A)-(C^{S}(l)+C^{CHD}(l)), \end{aligned}$$

for $$t=1, \dots ,T-1,l\in L,m\in M,$$ where

$$\begin{aligned} R(l,m)&= R_0(d^{S}(l))(d^{CHD}(l))(md^{ST}(A)),\\& \quad l\in L,~ m\in M \end{aligned}$$

describes the reward for 1 quality-adjusted life. The decrement factors $d^{S}, d^{CHD},$ and $d^{ST}$ denote the decrease in quality of life from a stroke (S), a coronary heart disease (CHD) event, or statins initiation (SI), respectively. The costs $C^{O}, C^{ST}, C^{S}$ and $C^{CHD}$, and $C^{FS}$ and $CF^{CHD}$ represent the cost of other health care for diabetes patients, cost of statin treatment, cost of initial hospitalization for stroke and CHD events, and cost of follow-up treatment for stroke and CHD events, respectively. This diabetes model suggested that initiation of treatment should be delayed in individuals predicted to have poor future adherence. Furthermore, the model predicted that over time, only 25% of patients will remain adherers for greater than 80% of the days during the study.

#### Modeling awareness

The effect of change in disease dynamics due to behavioral change and educational awareness have been modeled using ordinary differential equations-based models in several studies. Hallett et al. analyzed the effect of behavior change on the course of an HIV epidemic [62]. In their dynamic model, the behavior is incorporated by considering parameters such as mean rate of partner change and condom use in casual relationship as a step-function depending on time at which the change in behavior occurred and time it takes to reach a new value. In Mushayabasa et al., an ordinary differential equation model was used to quantify the role of an educational campaign in controlling Hepatitis C among women in prison [63]. Here, the effect of this campaign is reflected as an efficacy factor in conjunction to the parameters which represent the sharing of contaminated needles or syringes among the susceptible and exposed classes.

Ideas for VL modeling should also draw from modeling techniques used in economics in the context of social sciences to effectively optimize the cost and strategy in a resource-limited region. Fenichel et al. used an economic behavioral model in conjunction with the classical Susceptible-Infected-Recovered (SIR) model that explicitly models adaptive contact behavior [64]. The authors construct a utility function, an index which describes an individual’s well-being. The framework assumes that individuals make choices that maximize their utility. These decisions then impact disease risk, creating a feedback loop between disease risk and decisions made based on perceived disease risk. The authors demonstrate that fitting the classical SIR model to data generated by their new framework results in erroneous estimates of epidemiological parameters, because of its inability to jointly estimate behavioral and biological parameters. See Perrings et al. for a thorough review on the growing topic of economic epidemiology [65].

### Bridging the gap

**Fig. 3:**
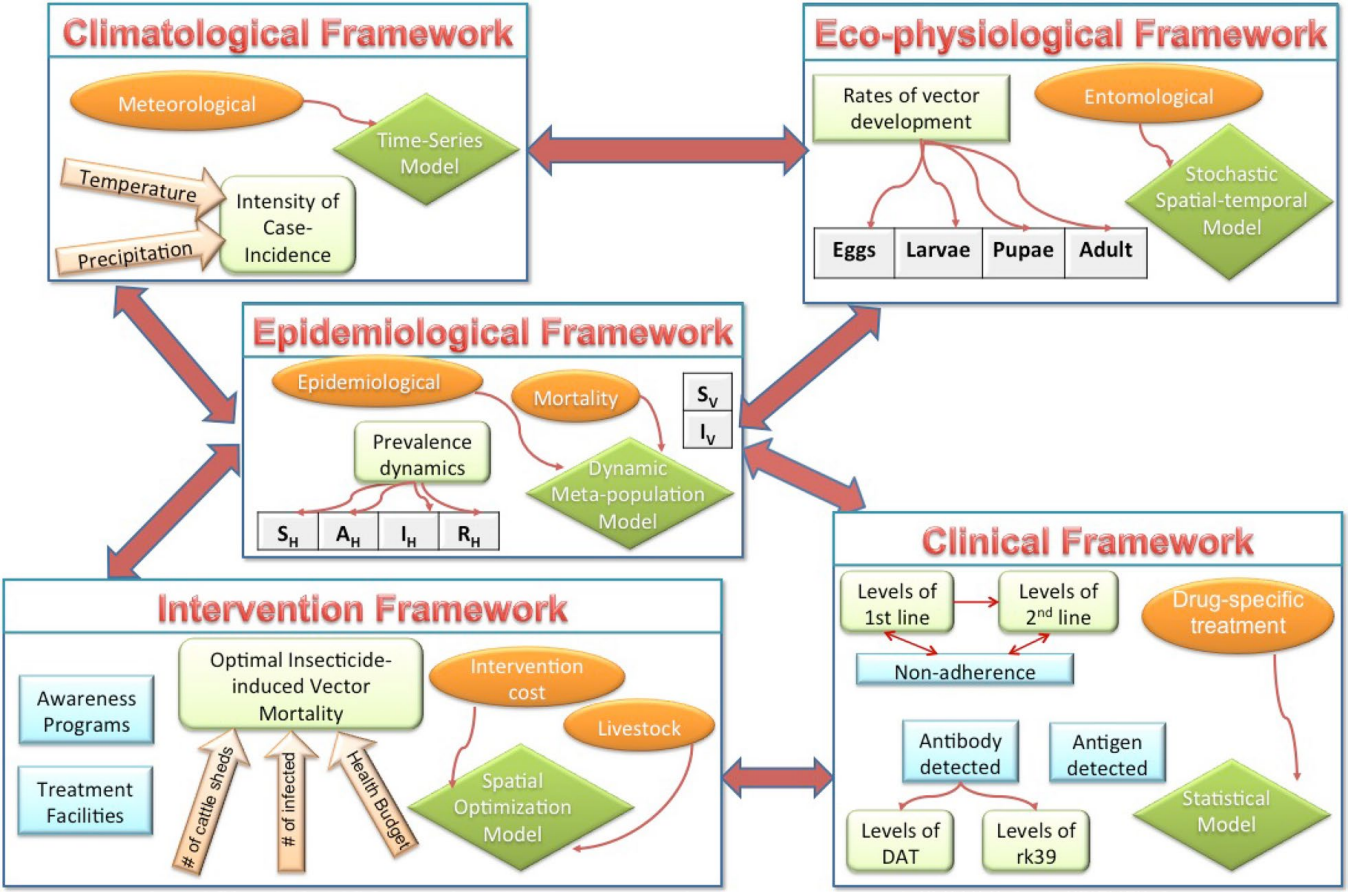
Caricature of critical categories of modeling frameworks for studying dynamics of vector-borne diseases (green rhombus), types of data needed in such frameworks (orange oval) and their potential links (red arrow). $S_V$ and $I_V$ represent densities of susceptible and infectious vectors. $S_H$, $A_H$, $I_H$ and $R_H$ are human epidemiological stages representing susceptible, asymptomatic, infectious, and recovered stages. 1st and 2nd line are for types of treatments whereas DAT and rk39 are for diagnostic methods. *Light green box* represents the output from respective modeling frameworks. These frameworks are mere examples in each categories and hence, each one of them can incorporate more details depending on the goals

The results of models addressing adherence to treatment, adaptive human behavior, and resource constraints emphasize the need to bridge the gap between socio-economic factors and existing mathematical modeling frameworks. Models of other vector borne diseases have captured some of the critical factors for visceral leishmaniasis (“Models of infectious diseases incorporating socio-economic risk actors” section). However, there is need for a more comprehensive set of frameworks that can incorporate local VL risk factors at multiple scales in the same dynamical model. The 6 A’s should be systematically incorporated into VL model frameworks to assess the sensitivity of VL dynamics to these six socio-economic factors. A schematic diagram depicting one such inter-dependent modeling framework is given in Fig. 3. Failing to address factors that result in significant changes in disease dynamics may result in models that do not effectively inform public health policy. Likewise, models that do not acknowledge resource constraints may lead to infeasible control policies.

## Discussion

NTDs, including VL, continue to spread in almost every continent on the planet, particularly in the poorest regions of modern human civilization [66, 67]. The campaign to eliminate VL from the Indian sub-continent, endorsed by the WHO, were set twice in the past (2010 and 2015) but target goals were not met [10]. The reasons for the ongoing spread and failure to control VL are thought to be mainly contributed by underreporting of cases [3], poor infrastructure, lack of awareness, poverty and inadequate control measures. In this review we have presented some of the less highlighted, but nonetheless critical factors/mechanisms, which are key in one of the worst VL affected impoverished regions, especially in the Indian state of Bihar. These factors may also play an important role in the transmission of other NTDs [3].

Mathematical models have been used to understand transmission dynamics of other tropical infections and strategize control measures under relevant constraints with varied success. Moreover, recent development of effective modeling techniques for infectious diseases, new sophisticated parameterizations methods [68] and useful validation tools have provided significant assistance to decision makers. However, modeling based study for evaluating temporal policies for NTDs are either limited or use risk factors that are known for well studied diseases. We suggest novel critical risk factors needed for effectively studying long term policies for NTDs and requires a rethinking of the manner in which address regional issues. There is a need for more modeling-based studies for NTDs, including for VL, that comprehensively address the six risk factors identified in this manuscript: Atmosphere, Access, Availability, Awareness, Adherence, and Accedence. Models that incorporate diverse local conditions as shown in Fig. 3 will allow formulation of effective public health strategies which will alleviate the burden of VL in under-developed regions like Bihar with it's resource constraints. Thus, we propose mathematical modeling as an efficient and cost-effective tool to devise meaningful control measures that will make the next WHO leishmaniasis elimination goal successful. Modeling studies are heavily dependent on the availability of region-specific surveillance, entomological and ecological data, and maintenance of a such database is also important for these regions where large scale integrated control programs are being introduced to achieve elimination.

In light of the major financial constraints in the affected regions, a hybrid dynamic optimization model (an example framework is shown in Fig. 3) will be necessary to explicitly calculate monetary (cost of interventions) and non-monetary (mortality and morbidity) factors related to VL. The building of such a model will require detailed quantification of every aspect of life in the regions, including non-tangible issues discussed here. Moreover, the execution of this model will require extensive sets of data on these varied aspects, which is challenging considering the current dearth of data. This will elucidate our understanding of the magnitude of this problem and estimate the relative importance of different socio-economic factors; thus, accurately predicting disease dynamics and informing effective public health policies.

## Additional file

**Additional file 1.** The process of the literature search as well as inclusion and exclusion of articles.

## Abbreviations

NTD: neglected tropical diseases
VL: visceral leishmaniasis
PKDL: post kala-azar dermal leishmaniasis
WHO: World Health Organization
DHS: District Health Society
PHC: Primary Health Center
NGO: Non-government Organizations
SSG: pentavalent antimonials
NVBDCP: National Vector Borne Disease Control Programme
PCR: polymerase chain reaction
DAT: direct agglutination test
LST: leishmanin skin test
SEI/SIR/SEIR: susceptible-exposed-infected-recovered model
IRS: indoor residual spraying
LLIN: long-lasting insecticide-treated nets
EVM: environmental management
HIV: human immunodeficiency virus
TB: tuberculosis
QALY: quality-adjusted life years
MDP: Markov decision process
CHD: coronary heart disease
