# Supplementary material for: Challenges in modeling complexity of neglected tropical diseases: a review of dynamics of visceral leishmaniasis in resource limited settings
Source: Emerg Themes Epidemiol. 2017 Sep 18;14:10. doi: 10.1186/s12982-017-0065-3 (PMC5604165; doi:10.1186/s12982-017-0065-3)
Supplement: Supplementary file 1 — Additional file 1. The process of the literature search as well as inclusion and exclusion of articles. [file 12982_2017_65_MOESM1_ESM.pdf]

Supplementary Document

The process of the literature search as well as inclusion and exclusion of articles.

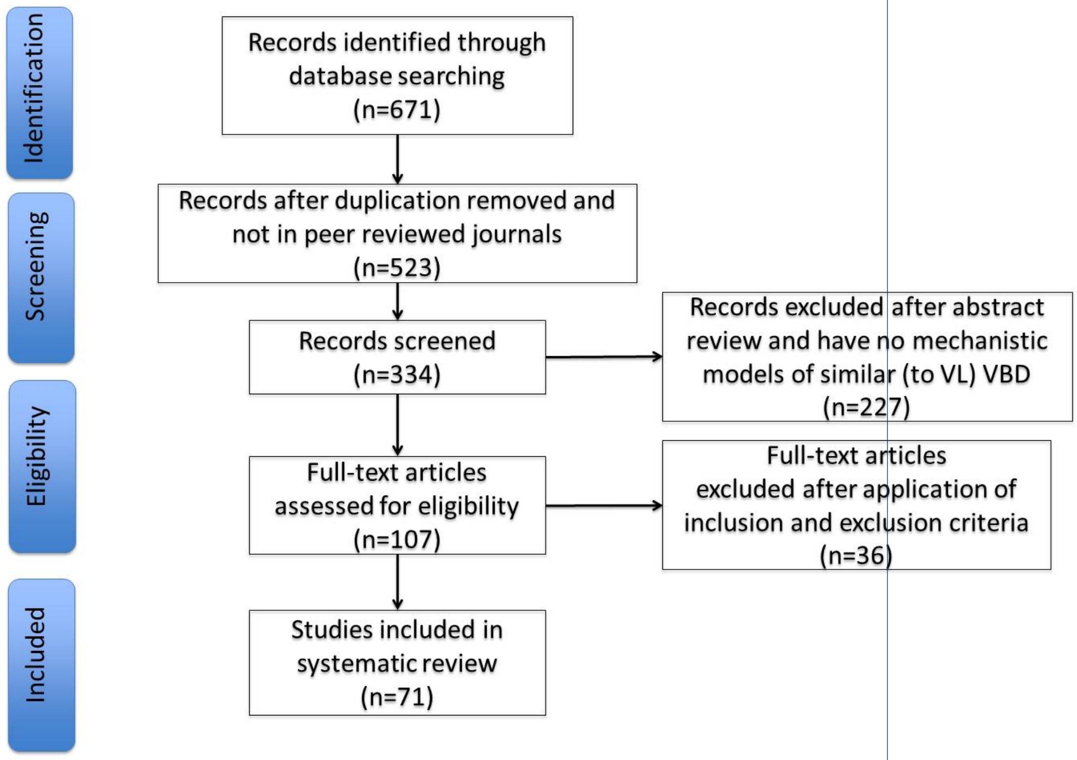

Figure 4: The process of the literature search as well as inclusion and exclusion of articles.
